# Whole-Genome Sequence of *Staphylococcus hominis* Strain J31 Isolated from Healthy Human Skin

**DOI:** 10.1128/genomeA.01548-16

**Published:** 2017-04-13

**Authors:** Rosanna Coates-Brown, Malcolm J. Horsburgh

**Affiliations:** Institute of Integrative Biology, University of Liverpool, Liverpool, United Kingdom

## Abstract

We report here the first whole-genome sequence of a skin-associated strain of *Staphylococcus hominis* determined using the PacBio long-read sequencing platform. *S. hominis* is a major commensal of the skin microflora. This genome sequence adds to our understanding of this species and will aid studies of gene traffic between staphylococci.

## GENOME ANNOUNCEMENT

*Staphylococcus hominis* is a consistent member of the human microflora, second in frequency among staphylococci to *Staphylococcus epidermidis* (1, 2). Despite its commensal status, *S. hominis* is an emerging clinical pathogen capable of causing infection in a variety of niches, such as the blood (3), particularly in the presence of long-term indwelling medical devices (4), and in the urogenital tract (5, 6). *S. hominis* is a reservoir for mobile genetic elements, such as the staphylococcal cassette chromosome *mec* element (SCC*mec*), which harbors the *mecA* gene for methicillin resistance (1). The presence of multidrug resistance phenotypes was identified in the population (7, 8), which is significant given the increasing rates of nosocomial *S. hominis* infections (7, 9, 10). Efforts to understand the role of *S. hominis* in human health and disease are significantly earlier in their infancy than those of *Staphylococcus aureus*, *S. epidermidis*, and *Staphylococcus haemolyticus*.

The costs of producing finished genomes from short-read sequencing data remain high despite the low cost per base offered by the technology. Long-read sequencing helps overcome the compromise between quality and cost (11). Benefits include the ease of genome assembly, together with increased resolution and accuracy, which aids our understanding of potential gene traffic between genomes (12).

*S. hominis* strain J31 was obtained from the volar forearm skin of a healthy volunteer in Liverpool, UK, in 2010; approval was granted by University of Liverpool Ethics Committee (RETH000089). PacBio sequencing libraries were prepared, and the genome was sequenced on the PacBio RS II platform. Contigs assembled using the PacBio distribution of the HGAP assembler with an *N*_50_ of 2,188,325 bp. The *S. hominis* J31 genome assembled into five contigs, the largest of which was 2,188,298 bp in length, with a coverage of 207×. Four smaller contigs were discrete plasmids. In total, the genome is 2,324,163 bp in length and, annotated using PROKKA, version 1.5.2 (13), was found to comprise 2,233 proteins, 70 tRNAs, and 23 rRNAs.

Long-read sequence data can help researchers fully exploit databases of short-read species-wide whole-genome sequence data. This sequenced genome of *S. hominis* J31 adds to the pool of species data and will help comparative genome studies of *S. hominis* and other staphylococci to increase understanding of the genetic repertoire at the genus and species levels. There is increasing importance on surveying the reservoir of genes that species of bacteria share in the skin niche, particularly gene flows into the pathogenic species *S. aureus* and intraspecific trait variation that structures communities (1, 14, 15).

### Accession number(s).

The draft genome sequence described in this paper is deposited in the ENA under the accession number FBVO01000000. The version described is the first version.
